# Comparison of total ionic strength adjustment buffers III and IV in the measurement of fluoride concentration of teas

**DOI:** 10.1177/0260106018758781

**Published:** 2018-04-04

**Authors:** Shilpa Patel, Narges Omid, Fatemeh V Zohoori, Anne Maguire, Kevin J Waldron, Ruth A Valentine

**Affiliations:** 1 Centre for Oral Health Research, Newcastle University, UK; 2 School of Health and Social Care, Teesside University, UK; 3 Institute for Cell and Molecular Biosciences, Newcastle University, UK

**Keywords:** Tea, fluoride, aluminium, TISAB, caffeine

## Abstract

**Background:**

Tea is the second most consumed drink in the UK and a primary source of hydration; it is an important source of dietary fluoride (F) for consumers and also abundant in aluminium (Al). Varying ranges of F concentrations in teas have been reported worldwide which may be, in part, due to differences in analytical techniques used to measure this ion.

**Aim:**

The effect of using total ionic adjustment buffers (TISAB) III or IV when measuring F concentration of black teas available in the UK was investigated and compared. Based on this evaluation, the effects of three different infusion times, 1 min, 10 min and 1 h, caffeine content and tea form on the F contents of the tea samples were investigated.

**Methods:**

The F concentrations of 47 tea samples were measured directly using a fluoride ion-selective electrode (F-ISE), TISAB III and IV and infusion times of 1 min, 10 min and 1 h.

**Results:**

Mean (SD) F concentration of tea samples for all infusion times was statistically significantly higher (*p* < 0.001) measured by TISAB IV (4.37 (2.16) mg/l) compared with TISAB III (3.54 (1.65) mg/l). A statistically significant positive correlation (*p* < 0.001) was found between Al concentration (mg/l) and differences in F concentration (mg/l) measured using the two TISABs; the difference in F concentration measured by the two TISABs increased with the magnitude of Al concentration.

**Conclusion:**

Due to higher concentrations of F and Al in teas and their complexing potential, use of TISAB IV facilitates more accurate measurement of F concentration when using an F-ISE and a direct method.

## Introduction

The tea plant is known to have a high bioaccumulation of both fluoride (F) and aluminium (Al), absorbing high levels from acidic soils at high temperatures. Both elements are accumulated mainly in leaves, with higher concentrations found in mature leaves. F accumulation has been reported to range from 300 to 1000 mg/kg in young leaves and from 600 to 2800 mg/kg in old leaves (Fung et al., 1999), while Al ion concentrations from 997 to 5600 mg/kg have been found in young and old leaves, respectively (Wong et al., 1998). Complexes of F with Al occur in acidic soils (pH < 5.5) and form AlF^2+^, AlF_2_^+^ or AlF_3_, with the latter being the dominant form (Fung et al., 1999).

In contrast to the concentrations found in tea leaves themselves, the amounts of F and Al ions released to tea liquors during infusion are much lower. For example, for dry tea products containing 300–2677 mg/kg Al and 107–878 mg/kg F, the concentrations in tea infusions were reported at 0.7–3.5 mg/l (Wong et al., 1998) and 0.6–1.9 mg/l (Fung et al., 1999).

Optimal exposure to F has benefits for oral health through prevention of dental caries and encouragement of bone and teeth remineralisation (Fung et al., 1999; McArthur, 2014; World Health Organization, 1994). These oral health benefits are best achieved through daily consumption of drinking waters with low F concentrations of 0.8–1.0 mg/l (World Health Organization, 2010). However, chronic excessive systemic (body) exposure to F can have health implications, including increased risk of dental fluorosis (mottled spots seen on tooth enamel) if the excessive exposure is during the ‘window of susceptibility’ of early childhood, and skeletal fluorosis, a crippling condition leading to an increase in bone mass if the excessive exposure extends into and through adulthood (Den Besten and Li, 2011; Joshi et al., 2011; Medical Research Council, 2002; World Health Organization, 1994). An upper tolerable intake of 10 mg of F per day for adults aged 19 years or above has been recommended with a dietary reference intake (DRI) of 4 mg/day (Otten et al., 2006).

Exposure to Al is limited but can occur from drinking waters, in which it is used as a coagulant to reduce organic matter, as well as from foods, usually due to Al-containing food additives and from antacids. Al has been suggested as a possible risk factor for dementia promotion, onset and progression (Dolara, 2014; Flaten, 2002; Henderson et al., 2002), but the evidence remains equivocal. The Joint Food and Agriculture Organization (FAO)/World Health Organization (WHO) Expert Committee on Food Additives and Food Contaminants has recommended a provisional tolerable weekly intake (PTWI) of Al of 1.0 mg/kg bw (FAO/WHO, 2006). The bioavailabilities of F and Al are dependent upon their chemical forms. Regarding F, its bioavailabilities in water, tea and solid foods differ, being up to 100% in water, 94.9% in teas (World Health Organization, 1984) and between 50% and 80% in solid foods, depending on their chemical composition (Grooper et al., 2009). The same trend is seen for aluminium, with systemic uptake of Al being generally very low, but higher from water (0.28% of intake absorbed) and from teas (0.37%) than from solid foods (0.1% of intake absorbed) (Dolara, 2014; Yokel and Florence, 2008). Since tea is the second most consumed drink worldwide after water, among both adults and children (Emekli-Alturfan et al., 2009), it may form a significant contribution to an individual’s F and Al intake. In view of this, many studies have measured F and Al contents of different types of teas worldwide.

Globally, black tea infusions have been reported to vary greatly in F content, ranging from 0.57 to 6.01 mg/l and in Al content, from 0.065 to 15 mg/l (Cao et al., 2006; Chan et al., 2013; Emekli-Alturfan et al., 2009; Flaten, 2002; Wong et al., 2003; Yi and Cao, 2008). These variations primarily reflect the local geological, cultivation and processing conditions as well as the different forms of teas and brewing times used. However, in addition, the varying ranges of F and Al concentrations reported may be, in part, due to differences in the analytical techniques used. For F these include ion chromatography (IC), spectrophotometry and fluoride ion-selective electrode (F-ISE), with the latter being used in the majority of studies. In contrast, for Al measurement the methods used range from graphite furnace atomic absorption spectrometry (GFAAS) and inductively coupled plasma atomic emission spectrometry (ICP-AES) to inductively coupled plasma mass spectrometry (ICP-MS), with ICP-AES being the primary method used (Flaten, 2002).

However, since the F-ISE responds only to free F ions, samples which contain free or ionisable F ions should be analysed directly after adding total ionic adjustment buffer (TISAB; Martínez-Mier et al., 2011). This buffer has an important role in reducing interference from certain elements (e.g. aluminium, iron and silicon). The purpose of adding TISAB to samples is threefold: (a) and (b) to adjust the sample and standards to the same ionic strength and pH; and (c) to de-complex F ions from certain interfering elements. TISABs are commonly used in their commercially available forms, namely II, III and IV. TISAB II and III adjust the pH of samples and standards to between 5 and 5.5, while TISAB IV adjusts to a pH of approximately 8.5. All TISABs contain an Al ion masking agent, cyclohexylenedinitrilotetraacetic acid (CDTA), which complexes with Al preferentially, freeing F from complexes with this ion (Schamschula et al., 1985; Tušl, 1970). TISAB II and III can complex approximately 5 mg/l of Al in a sample containing 1 mg/l of F, and the ratios used to buffer the samples are 1:1 and 1:10 for TISAB II and III respectively, while TISAB IV is recommended for use in samples with higher concentrations of Al ion since it contains stronger chelating agents and eliminates interference from Al–F complexes (Thermo Scientific, Orion, User Guide 2014; Caslab 1996). According to the literature, the most commonly used method for F analysis of teas is a direct method (Cao et al., 2006; Chan et al., 2013; Emekli-Alturfan et al., 2009; Fung et al., 1999; Koblar et al., 2012; Malinowska et al., 2008). However these studies have used TISAB II (Chan et al., 2013) or III (Emekli-Alturfan et al., 2009), or have not reported the type of TISAB used (Cao et al., 2006; Emekli-Alturfan et al., 2009; Fung et al., 1999; Koblar et al., 2012; Malinowska et al., 2008). In view of the high Al concentrations of up to 13 mg/l reported for tea samples (Erdemoglu and Gucer, 2005; Koch et al., 1988), measurements of F in teas reported in the literature might be underestimated due to types of TISABs used, which do not allow for the high Al content of some teas.

These high concentrations of both F and Al in teas, as well as the wide range of F concentrations in teas reported in the literature, highlight the importance of using standardised measurement procedures when using a F-ISE and a direct method. Therefore the main aim of this study was to investigate and compare the effect of using two different TISABs; III and IV, when measuring F concentration in black teas, commercially available in the UK, taking into account the Al concentrations. Based on the preferred/appropriate TISAB, the subsidiary aim was to investigate the effect of three different infusion times, 1 min, 10 min and 1 h, as well as the caffeine content and form of tea on the F concentrations of the tea samples

## Method

### Sourcing samples

In total, 47 black teas were identified through a thorough search of supermarket websites and/or visiting the supermarkets directly. The teas were purchased from the following UK supermarkets, Asda, Morrisons, Sainsbury’s and Tesco in March 2014 and samples categorised based on: (a) caffeine content, caffeinated or decaffeinated, and (b) type of packaging, teabag, loose (tea leaves) and instant tea.

### Sample preparation

Tea samples were prepared to represent infusions ready for consumption, simulating the customary habits of tea drinkers (Ruxton and Hart, 2011). A longer non-representative time point of 1 h was also included to look at a maximal effect of infusion on F content. For tea bags and loose teas, where the infusion times could be measured, infusion times of 1 min, 10 min and 1 h were used.

Tea bags: Three whole tea bags (one for each time point) were separately infused with 200 ml of boiling double de-ionised water (DDiH_2_O) for 1 min, 10 min and 1 h; tea bags were not squeezed or stirred.

Loose tea: Manufacturers’ recommendations of 1 level teaspoon (2.20 g) of dry leaves per cup were followed and 200 ml boiling DDiH_2_O added prior to three separate infusions timed at 1 min, 10 min and 1 h. The infusions were then strained with a stainless steel tea strainer, removing loose leaves to prevent further infusion and the strainer rinsed with DDiH_2_O twice between each use.

Instant tea: Product recommendations of 1 teaspoon per cup (1.00 g) were used. Due to the tea form, an infusion time was not applicable, therefore one infusion was prepared per sample. One gram of tea granules was weighed and 200 ml boiling DDiH_2_O added.

All prepared tea liquors were cooled to room temperature, stirred and transferred to 30 ml polystyrene universal containers. For F analysis the infusions were freshly prepared on a daily basis and analysed on the same day. For Al analysis, samples were mixed thoroughly and frozen prior to analysis.

### F analysis of tea liquors

Tea samples were analysed for F by a direct method (Martínez-Mier et al., 2011) and in triplicate using a F-ISE (Model 9409; Orion) and meter (Model 900A; Orion) after sample buffering with either TISAB III (Orion 940911 Thermo Scientific USA) or TISAB IV (Fluka analytical, Sigma-Aldrich, Germany). The validity of the method was determined by adding a known amount of F to 10% of both the TISAB III and TISAB IV buffered samples analysed and measuring the F recovery. In addition, 10% of all tea samples were re-analysed on a different day to check the reliability of the method.

### Determination of Al content

All frozen tea liquor samples were thawed to room temperature before analysis. ICP-MS was used to determine the Al content of the tea liquors using a Thermo X-series instrument operating in standard mode. For each analysis, 500 µl of tea sample was diluted 10-fold with 2.5% (w/v) high-purity HNO_3_ (Merck) containing 20 µg/l silver (Ag) and platinum (Pt) as internal elemental standards. For each sample, aluminium (^27^Al), silver (^107^Ag) and platinum (^195^Pt) isotopes were monitored sequentially using the peak-jump method (100 individual reads of 20 milliseconds on each isotope, across three channels of 0.02 atomic mass unit (AMU) separation, each in triplicate). Al concentrations were determined by comparison with matrix-matched standard solutions of known elemental composition (0, 1, 5, 10, 50, 100, 500, 1000 and 5000 mg/l) analysed within the same run.

### Data handling and statistical analysis

To allow comparison of the performance of TISAB III and IV at lower and higher Al concentrations, F data were grouped according to the Al concentration of the samples; <5 mg/l and >5 mg/l. Data were analysed using SPSS version 22 (SPSS, Chicago, IL, USA). Descriptive analysis was carried out to report mean (SD) and median (maximum and minimum) values to allow comparison with other studies. Data were treated according to the results of their normality testing. A paired t-test was performed to compare all F concentrations measured by TISAB III and IV (*n* = 137). Two-way analysis of variance (ANOVA) with Sidak’s multiple comparison test was used to compare F concentration of tea samples between the two types of TISAB used. Regression analysis was used to investigate the effect of Al concentration on the difference between measured F concentration using TISAB III and IV. Mann–Whitney U-tests were used to investigate the effect of caffeine content and tea form on the F concentration of tea samples and a Kruskal–Wallis test, followed by pairwise multiple comparison analysis was performed to investigate the effect of infusion time on F concentration.

## Results

The mean (SD) recovery of F added to a randomly selected sub group of 10% of tea samples was 98.5 (3.1) % and 98.0 (2.9) % using TISAB III and TISAB IV, respectively, indicating satisfactory validity of the F analysis method used. In addition, the reliability of the method was checked by re-analysis of 10% of tea samples using both TISAB III and IV, which showed <4% difference between analysis and immediate re-analysis. A small number of tea samples were analysed immediately after infusion and again after 24 h and 48 h of storage at 4°C. Samples were incubated in the fridge and analysed at room temperature. Analysis with TISAB III resulted in a significant decrease in F concentration 48 h post preparation. Re-analysis with TISAB IV after 48 h storage showed no significant change in F concentration with time (results not shown). The reduction in F concentration measured with TISAB III is most probably due to the phenomenon of ‘tea creaming’ (Jobstl et al., 2005), a naturally occurring precipitation effect that occurs during cooling due to some of the components that are soluble in hot water, being insoluble in cold water and precipitating (approximately 30% of the total solids). Due to the possible ‘creaming’ effect after sample storage at 4°C, teas were analysed immediately after tea liquor sample preparation. As Table 1 shows, mean (SD) F concentration (mg/l) for 45 teas measured at all three infusion times and the two instant tea samples measured at one infusion time (*n* = 137) by TISAB III and IV were 3.54 (1.65) and 4.37 (2.16) mg/l, respectively. A statistically significantly (*p* < 0.001) higher F concentration was measured when TISAB IV was used.

**Table 1.** Mean (SD) F concentration (mg/l) of 45 tea samples measured at all three infusion times plus two instant tea samples measured at one infusion time (*n* = 137) using TISAB IV and III and mean (SD) difference in measured F concentration between buffers.

Mean (SD) F concentration (mg/l) measured by using		Difference in measured F concentration (mg/l)*	*p* value
TISAB IV	TISAB III		
4.37 (2.16)	3.54 (1.65)	0.83 (1.03)	<0.001

*TISAB IV − TISAB III.

**Figure 1.**
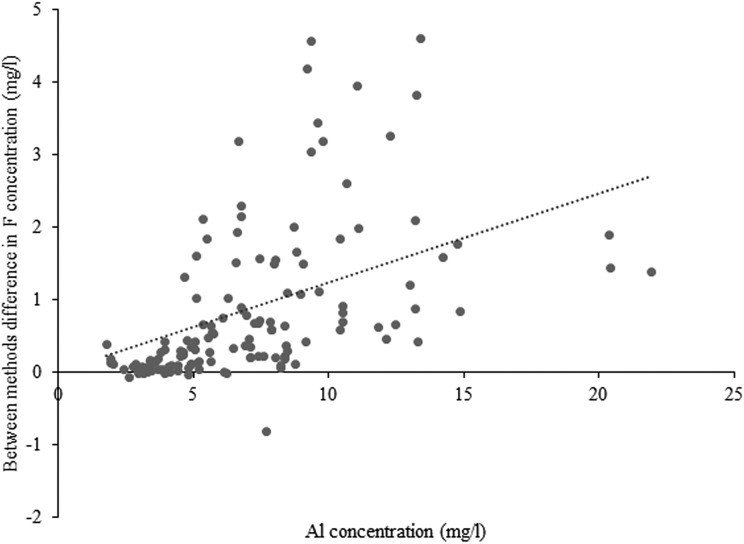
The effect of Al concentration (mg/l) on the difference between measured F concentration (mg/l) using TISAB III and IV. Equation for best-fit line: between-method difference in F concentration (mg/l) = 0.123 × Al concentration (mg/l).

A statistically significant positive correlation (R^2^ = 0.2666, *p* < 0.001) was found between Al concentration (mg/l) and the differences between F concentration (mg/l) measured using the two TISABs (Figure 1) with ‘between-method differences in measured F concentration (mg/l) = 0.123 × Al concentration (mg/l)’. This highly significant correlation suggests that the difference in F concentration measured by the two TISABs increased with the magnitude of Al concentration, i.e. an increase in the difference in F concentration measured between the two TISABs of 0.123 mg/l for every 1 unit increase in Al concentration (mg/l).

**Figure 2.**
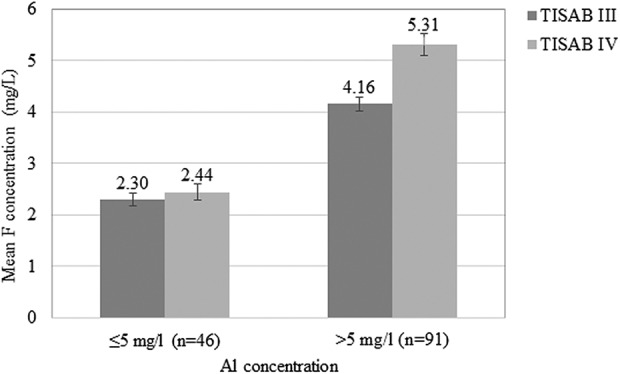
Comparison of mean measured F concentration (mg/l) using TISAB III and IV according to the Al concentration (<5mg/l and >5mg/l) of the samples.

Figure 2 compares the mean measured F concentration (mg/l) using TISAB III and IV according to the Al concentration (<5 mg/l and >5 mg/l) of the samples. For tea samples with a Al concentration of <5 mg/l, the difference in mean F concentration (mg/l) measured using TISAB III and IV was low (mean (SD) F concentration: 2.30 (0.86) mg/l and 2.44 (0.89) mg/l, respectively; ns), whereas for tea samples with a Al concentration of >5 mg/l, the corresponding difference was relatively high (mean (SD) F concentration: 4.16 (1.62) mg/l using TISAB III and 5.33 (1.98) mg/l using TISAB IV; *p* < 0.001).

Since the measured F concentrations were statistically significantly higher in samples analysed using the TISAB IV buffer compared with TISAB III, the TISAB IV was our preferred buffer for the F analysis of tea and therefore only analyses and comparisons of teas for TISAB IV based samples are presented in the following sections.

Information on the brand/names, form of teas (e.g. loose, tea bags and instant), caffeine content and F concentration of all tea samples at infusion times of 1 min, 10 min and 1 h is presented in Table 2. The majority of the 47 analysed tea samples were caffeinated (*n* = 39) and in the form of teabags (*n* = 37). The F concentrations of the two instant teas were 7.78 and 9.91 mg/l for PG Tips and Tetley pure tea granules respectively. Among the remaining samples, the highest F concentration (9.44 mg/l) was found for PG Tips (decaffeinated) after 1 h infusion, and the lowest (1.06 mg/l) for Twinings Assam (caffeinated) after 1 min of infusion.

**Table 2.** Information on name/brand, form of packaging (B = tea bag form; L = loose tea form; I = instant tea form), caffeine content (C = caffeinated; D = decaffeinated), country of origin and F concentrations (mg/l) at infusion times of 1 m, 10 m and 1 h for 47 black tea samples.

Product name/brand	Tea form^a^ / caffeine content^b^	Country of origin	F concentration (mg/l) measured by TISAB IV		
			1 min	10 min	1 h
Asda Chosen by You	B/C	Data unavailable	3.44	6.00	6.32
Lancashire Tea Standard Blend	B/C	Data unavailable	3.88	5.27	7.30
Morrisons Red Label	B/C	Data unavailable	3.67	4.41	6.05
PG Tips	B/C	Kenya	6.43	8.41	8.96
Red Label by Sainsbury’s	B/C	India, Kenya, Malawi, Sri Lanka	3.19	4.08	5.34
Tesco Original Medium Strength Tea	B/C	Data unavailable	4.20	6.41	6.86
Tetley Original	B/C	China, India, Sri Lanka	5.40	8.42	8.41
Twinings Original English Breakfast	B/C	Kenya, India, Sri Lanka	1.65	3.23	5.14
Typhoo	B/C	China, Kenya, India	4.64	6.01	6.29
Yorkshire Tea	B/C	Kenya, India	2.16	2.58	6.13
Asda Smart Price Tea Bags	B/C	Data unavailable	3.23	5.90	8.40
Morrisons Savers	B/C	Data unavailable	3.33	5.05	5.33
Sainsbury’s Basics	B/C	India, Kenya, Malawi, Sri Lanka	4.32	7.07	7.08
Tesco Everyday Value	B/C	Data unavailable	4.75	7.79	8.95
PG Tips the Fresh One	B/C	Kenya	4.37	6.84	7.06
Twinings the Everyday Tea	B/C	China, India, Kenya, Indonesia	2.98	4.02	5.07
Sainsbury’s Taste the Difference Earl Grey	B/C	India, Kenya, Malawi, Sri Lanka	3.65	3.86	3.01
Twinings the Earl Grey	B/C	China	3.01	4.51	4.61
Tetley Earl Grey	B/C	China, India, Sri Lanka	3.51	4.65	5.09
Asda Chosen by You Assam	B/C	India	1.59	2.62	2.74
Asda Chosen by You Ceylon	B/C	Sri Lanka	1.32	1.80	2.07
Tesco Finest Assam	B/C	India	1.28	1.71	1.89
Twinings Assam	B/C	India	1.06	1.38	2.05
Twinings Ceylon	B/C	Sri Lanka	1.20	1.55	1.69
Morrisons Extra Strong	B/C	Data unavailable	2.96	4.34	4.48
PG Tips the Strong One	B/C	Kenya	5.31	7.40	7.64
Extra Strong Red Label by Sainsbury’s	B/C	India, Kenya, Malawi, Sri Lanka	1.96	3.32	3.62
Tetley Extra Strong	B/C	China, India, Sri Lanka	2.12	4.00	4.15
Yorkshire Gold	B/C	India, Rwanda	1.44	1.95	2.86
Asda Chosen by You Decaffeinated	B/D	Data unavailable	2.94	3.00	4.16
Morrisons Decaffeinated	B/D	Data unavailable	3.16	5.52	6.05
PG Tips Decaf	B/D	Kenya	6.77	9.28	9.44
Decaffeinated Red Label by Sainsbury’s	B/D	India, Kenya, Malawi, Sri Lanka	2.72	5.05	5.25
Tesco Decaff Medium Strength Tea	B/D	Data unavailable	3.04	4.75	5.80
Tetley Decaf	B/D	China, India, Sri Lanka	4.99	7.69	8.66
Typhoo Decaf	B/D	China, Kenya, India	3.25	4.55	5.33
Yorkshire Tea Decaf	B/D	India, Kenya	3.06	7.72	9.01
Asda Chosen by You Loose Leaf Tea	L/C	Data unavailable	2.42	2.96	2.82
Morrisons Red Label Loose Tea	L/C	Data unavailable	2.65	3.11	3.25
PG Tips Loose Tea	L/C	Kenya	3.92	4.79	4.56
Red Label Loose Tea by Sainsbury’s	L/C	India, Kenya, Malawi, Sri Lanka	2.16	2.48	2.66
Tesco Original Loose Leaf	L/C	Data unavailable	3.03	3.66	4.28
Twinings Original English Breakfast Loose	L/C	India, Kenya, Sri Lanka	1.46	1.86	1.62
Typhoo Leaf Tea	L/C	China, Kenya, India	1.88	2.29	2.34
Yorkshire Tea Leaf Tea	L/C	India, Kenya	2.04	2.51	3.03
PG Tips Pure Tea Granules	I/C	Kenya	7.78		
Tetley Pure Tea Granules	I/C	China, India, Sri Lanka	9.91		

Based on infusion time, the mean (SD) F concentration of tea samples infused for 1 h was higher (5.17 (2.27) mg/l) than those infused at 10 min (4.52 (2.11) mg/l) and 1 min (3.16 (1.36) mg/l), and the difference between infusions at 1 min and 10 min as well as those infused at 1 min and 1 h was statistically significant (*p* < 0.001) (Table 3). Data for instant teas are presented separately since they could only be prepared at a single time point.

**Table 3.** Median (range) and mean (SD) F concentration (mg/l) of teas (*n* = 45; excluding instant tea) at three infusion times, 1 min, 10 min and 1 h, using TISAB IV.

Infusion time	F concentration (mg/l)				
	Median	Minimum	Maximum	Mean	SD
1 min (*n* = 45)	3.04	1.06	6.77	3.16^a^	1.36
10 min (*n* = 45)	4.41	1.38	9.28	4.57^a^	2.11
1 h (*n* = 45)	5.14	1.62	9.44	5.17^a^	2.27
All (*n* = 135)	3.99	1.06	9.44	4.29	2.12

^a^Statistically significant difference *p* < 0.001 (Kruskal–Wallis test, pairwise multiple comparisons analysis).

Based on caffeine content, across the three infusion times the F concentrations of decaffeinated teas were significantly higher (*p* < 0.05) than caffeinated teas (Table 4). On the same basis, across all infusion times and as Table 4 also describes, a significantly (*p* < 0.001) higher F concentration was found in teabags (4.62 (1.86) mg/l) compared with loose teas (2.82 (0.87) mg/l).

**Table 4.** Median (range) and mean (SD) F concentration (mg/l) of 45 tea samples (excluding the two instant teas) based on caffeine content and tea form across all three infusion times (total number of samples =135).

Sample category	F concentration (mg/l)					
	Median	Minimum	Maximum	Mean	SD	Mann–Whitney U
Caffeine content: Caffeinated (*n* = 111) Decaffeinated (*n* = 24)	3.93 4.72	1.06 2.72	8.96 9.44	4.04 5.47	1.80 1.74	*p* < 0.05
Tea form: Loose tea (*n* = 24) Teabags (*n* = 111)	2.63 4.42	1.65 1.48	4.42 8.50	2.82 4.62	0.87 1.86	P < 0.001

## Discussion and conclusion

This study measured the F concentration of a range of teas available in the UK market using a direct method and F-ISE. The main aim was to investigate and compare the effect of using two different TISABs, III and IV.

According to the literature, the preferred method for ionic F analysis of teas is a direct method using F-ISE (Cao et al., 2006; Chan et al., 2013; Chandrajith et al., 2007; Emekli-Alturfan et al., 2009; Fung et al., 1999; Koblar et al., 2012; Malinowska et al., 2008). Although an overnight acid-diffusion pre-treatment can be used for samples containing non-ionic F complexes, which releases F ions from such complexes prior to measurement using F-ISE (Soto-Rojas et al., 2005; Taves, 1968), the use of a direct method was preferred. This is mainly because >90% of F in tea infusions exists in the ionic form which can be measured directly after adding a TISAB buffer (Fung et al., 1999; World Health Organization, 1984). The majority of studies have used a TISAB, with sample ratio of 1:1, and most studies have primarily used TISAB II. However, the reported high levels of Al in tea samples, and the complexing interaction of Al with F, suggest that these studies may have underestimated F concentrations in view of the specific TISABs used. The statistically significantly higher concentration of F measured when using TISAB IV compared with TISAB III shows that TISAB IV is more capable of releasing F ions due to the reduction in the stability of the Al-F complex through adjustment of the sample pH to 8.5.

The effect of TISAB IV in releasing a greater proportion of the F ions was further confirmed with the statistically significant positive correlation (*p* < 0.001) found between Al concentration (mg/l) and difference in F concentration (mg/l) measured using the two TISABs. The study found an increase in the difference in F concentration of >0.1 mg/l for every 1 unit increase in Al concentration (mg/l). The manufacturer’s recommended limits of measurement when using TISAB III at 1 ppm F concentration are 5 mg/l Al (Thermo Scientific Orion ion plus Fluoride Electrode, Instruction Manual, 2014). Interestingly, in these tea samples, all but one contained more than 1 ppm F (1 mg/l), indicating that TISAB III may not be a suitable buffer to measure F in tea infusions, even with relatively low Al concentrations.

Previous reports have determined F concentrations of black tea infusions ranging from low levels identified in Brazilian tea samples (0.13 mg/l) (Hayacibara et al., 2004) to high levels measured in UK sourced teas (8.85 mg/l); both studies used ISE with TISAB II (Chan et al., 2013). The F concentrations found in teas purchased in the UK for this present study fit within and extend beyond this range, but the results obtained are in agreement with previous outcomes; that F does accumulate in the tea leaves and that its content in tea liquors increases with the duration of infusion (Chan et al., 2013; Koblar et al., 2012; Quock et al., 2012). However, the statistically significant positive correlation observed between F and Al concentrations of the teas analysed highlights the importance of using the correct buffer prior to measurement. Although F in tea has been extensively investigated and analysed, the range of concentrations previously proposed may be lower than the actual total available F concentrations (i.e. the ionic F content available for absorption in the gastrointestinal tract (GIT)) present in black teas. Concentrations of F in tea are dependent upon the original source of the tea plant as a raw material, its maturity and the pH of the soil in which it has grown (Janiszewska and Balcerzak, 2013). Many popular brands and supermarket varieties of teas are produced through mixing of *Camellia sinensis L.* blends derived from various geographical locations (Hayacibara et al., 2004) (Table 2). Pure blends from Assam and Ceylon had the lowest F concentration in comparison with all other teas, ranging from 0.94 to 1.32 mg/l for tea liquors. These data are also in line with studies analysing similar pure blend teas in the UK and Europe, prepared with shorter infusion times (Chan et al., 2013; Chandrajith et al., 2007) as well as the upper limit ranges found in Ceylon tea analysis (0.32–1.68 mg/l, respectively) (Chandrajith et al., 2007). Reports in the UK and China have suggested that economy blend teas and teas with no claim of tea plant country of origin have the highest concentrations of F (Cao et al., 2006), and previous studies focusing on economy teas in the UK support this, recording the F concentration of economy ‘value’ teas to range from 6.20 to 8.85 mg/l (Sainsbury Basics and Tesco Value, respectively) when infused for up to 30 min (Chan et al., 2013). These results are in agreement with the current study in which the F content of economy teas ranged from 2.88 to 9.00 mg/l at 1 h infusion, and were some of the highest F concentrations recorded. Economy branded teas are manufactured with older leaves, branches and roots of the tea plant (Wong et al., 2003); these mature parts of the tea plant are estimated to contain up to 10-fold more F than the younger leaves and buds (Fung et al., 1999).

Previous studies investigating Al content independently, as well as investigations of F and Al contents together, have found the Al content in black teas to range from 0.065 (Rao and Rao, 1994) to 13 mg/l (Erdemoglu and Gucer, 2005; Koch et al., 1988). These studies applied different measurement methods including atomic emission spectrometry (AES), ICP-AES, flame atomic absorption spectrometer (FAAS), GFAAS and ICP-MS. In the present study ICP-MS was used and found the Al concentration for the combined infusion times (*n* = 137) ranged from 1.7 to 21.9 mg/l which is wider than the 1–6 mg/l previously reported by other studies (Flaten, 2002). This may partly be explained by differences in the analytical methods used, for example, a previous study investigating Al content by ICP-AES of Tetley black tea infused for 3 min in 250 ml boiling deionised water found 2.90 mg/l (Powell et al., 1993) while the current study determined Al concentration at 7.46 mg/l in Tetley Original tea bags infused for 1 min. Another possible explanation for this variability may be the year to year variation in multiple crops used to blend this tea. ‘Tea creaming’ may also influence levels of Al measured in teas, although the addition of HNO_3_ to the sample in a digestion step prior to ICP-MS analysis should have allowed Al to be released from any insoluble material and measured. The higher concentrations determined in this study may be indicative of more sensitive detection limits of the ICP-MS in comparison with methods used in previous studies, or may be explained in some part by the variation between different batches used to blend the tea product.

In terms of the effect of infusion time on F content of teas, the current study used different infusion times of 1 min, 10 min and 1 h to cover the range of infusion times previously measured by other studies (up to 10 min), as well as to mirror customary home tea preparation (shortened infusion time of 1 min). A longer non-representative time point of 1 h was included to look at a maximal effect of infusion of F content.

A wide range of infusion times, between 2 min (Chan et al., 2013; Wong et al., 2003) and 6 h (Fung et al., 1999; Wong et al., 2003), has been reported in the literature for the preparation of tea infusions, with the resultant mean F concentrations ranging from 0.71 mg/l (Chan et al., 2013) to 1.89 mg/l (Fung et al., 1999) for samples prepared at 2 min and 6 h, respectively. The widest range in F concentrations was reported by Chan et al. between 0.71 and 8.80 mg /l for samples infused for 2 min and 30 min, respectively (Chan et al., 2013). However, another study, despite using a wider infusion time range (up to 6 h), reported a narrower range of F concentrations, between 0.91 mg/l at 5 min and 1.90 mg/l at 2 h time points (Fung et al., 1999). That range was smaller than the 1.06 mg/l to 9.44 mg/l range observed in this present study for samples infused at 1 min and 1 h, respectively, which are more in line with the range reported by Chan et al. (2013) in which a significantly higher F concentration was recorded after 30 min infusion.

It has been reported that during the first 30 min of infusion a large amount of F is released into the tea liquor, with 77–85% being released in the first 5 min and the amount of released F is directly proportional to the duration of infusion (Fung et al., 1999). The findings of the present study show that 61% of F was released in the first 1 min of infusion, and 88% in the first 10 min, with the amount of measured F statistically significantly increasing between 1 min and 10 min and between 1 min and 1 h, while the difference between 10 min and 1 h was not statistically significant.

In terms of the caffeine content of the teas, statistically significantly higher F concentrations were found in decaffeinated brands compared with caffeinated varieties, a finding previously reported along with one possible explanation; the use of fluoridated water in the decaffeinating process (Chan and Koh, 1996; Yi and Cao, 2008). This process involves removing caffeine from the tea by soaking the tea leaves in hot water for a period of time, filtering the solution through carbon to remove caffeine and returning the water to the tea for reabsorption of flavours and oils, however this is not generally used commercially. Another possible explanation is the known effect of caffeine on tea creaming. Tea with lower levels of caffeine produce less ‘creaming’ (Jobstl et al., 2005), and therefore potentially a lower loss of minerals during the cooling process, which may account for some of the variation observed in this study.

Fermentation is a vital stage in black tea manufacture, and is also characteristic of teas with elevated F concentrations (Janiszewska and Balcerzak, 2013; Malinowska et al., 2008). It has been suggested that this may be due, in part, to greater amounts of polyphenols (enhancing fermentation) present in the tea plant, however it has also been shown that polyphenols correlate negatively with F concentrations, with older plants having lower polyphenol but higher F concentrations (Lu et al., 2004). With regard to instant teas, freeze dry processing involves concentrating the extract from brewed pure tea leaves to produce a powdered form. The highly concentrated nature of the tea extract itself is the probable reason for the highest F concentrations being determined in the instant tea infusions when compared with loose tea and tea bags at 2.66 mg/l and 4.65 mg/l, respectively.

Regarding diet, the systemic bioavailability of F is considered to be high, between 50-80% in the fed state and 100% in a fasted state (Ophaug, 1990). F presents a biphasic nature in the form of benefits and risks to dental and general health and as a source of topical F in the mouth and systemic F when absorbed from the GIT (Simpson et al., 2001). Tea, together with dentifrices such as toothpaste and fluoridated water, could have both positive and negative health benefits depending on the overall level of consumption. Once absorbed systemically, 90% of the F retained in the body is incorporated into calcified tissues including bone and enamel crystallites (World Health Organization, 1994). The presence of Al reduces F bioavailability, while a deficiency in Al may heighten F uptake from the GIT (Den Besten and Li, 2011). The Al content of black teas, together with the positive correlation between F and Al concentrations, may reduce the body’s ability to absorb and utilise the F that tea infusions contain. In terms of F, a DRI of 4 mg/day and upper tolerable intake of 10 mg/day for adults aged 19 years and above has been recommended (Otten et al., 2006) and according to the UK National Diet and Nutrition Survey (Henderson et al., 2002) the average daily consumption of tea in the UK is 540 ml (2–3 servings). This indicates that, based upon the F content of teas found in the present study, levels of daily F consumption in a UK population living in optimally fluoridated areas could be towards the upper tolerable range. The current in vitro study used de-ionised water to prepare infusions, in contrast to the situation found in vivo where higher levels of F may be consumed in those areas where fluoridated water is available and used for tea making (Koblar et al., 2012).

Since teas may comprise a significant proportion of individual’s daily fluid intake, it is important to increase public awareness on any health implications of drinking excessive amounts of tea, when informing on the need for balance between benefit and risk. In addition, health professionals should take tea consumption among young children into consideration prior to prescribing F supplements. It is noted by the authors, however, that there is no evidence in Westernised society that consuming commercially available black tea increases the risk of developing fluorosis.

Due to the high concentration of F and Al in the tea samples analysed and their potential for complexing with F, the use of TISAB IV provides a more accurate measure of F concentration when using a direct method and F-ISE.

The wide range of F concentrations found in popular teas available in the UK together with fluoridated tap water being available for the preparation of tea infusions in some areas, highlights the need for further information on the typical amounts of F consumed through this beverage.
